# Immunological Significance of the ICI–PIT–ICI Sequence in Recurrent Oral Cancer: A Narrative Review with Illustrative Cases

**DOI:** 10.3390/diagnostics16142164

**Published:** 2026-07-10

**Authors:** Taiki Suzuki, Kenichi Kumagai, On Hasegawa, Taro Okui, Reo Aoki, Koichiro Kato, Chieko Masuda, Yoshihiro Ohashi, Yoshiki Hamada, Akihisa Horie

**Affiliations:** 1 Department of Oral and Maxillofacial Surgery, Kanto Rosai Hospital, Kawasaki 211-0021, Kanagawa, Japan; kumagaik-ora@h.u-tokyo.ac.jp (K.K.); taro1003@fujita-hu.ac.jp (T.O.);; 2 Department of Oral-Maxillofacial Surgery and Orthodontics, The University of Tokyo Hospital, Tokyo 113-8655, Japan; 3 Department of Oral and Maxillofacial Surgery, Tokyo Medical University Hachioji Medical Center, 1163 Tate-machi, Hachioji 193-0998, Tokyo, Japan; on-h@tokyo-med.ac.jp; 4 Department of Oral and Maxillofacial Surgery, School of Dental Medicine, Tsurumi University, 2-1-3 Tsurumi, Yokohama 230-8501, Kanagawa, Japan; hamada-y@tsurumi-u.ac.jp

**Keywords:** oral cancer, photoimmunotherapy, immune checkpoint inhibitor, sequential immunotherapy

## Abstract

Immune checkpoint inhibitors (ICIs) have improved clinical outcomes in recurrent or metastatic head and neck squamous cell carcinoma (HNSCC), including oral squamous cell carcinoma (OSCC). However, many patients eventually develop resistance to systemic therapy, highlighting the need for novel strategies that can restore or sustain antitumor immunity. Near-infrared photoimmunotherapy (PIT) has emerged as a tumor-selective locoregional treatment that not only induces targeted tumor cell death but also promotes antitumor immune activation through immunogenic cell death. This narrative review summarizes current evidence regarding PIT for recurrent oral cancer and explores the immunological rationale for sequential ICI–PIT–ICI therapy (ICI–PIT–ICI sequence). Within this framework, PIT-induced tumor antigen release and inflammatory activation may reinitiate elements of the cancer-immunity cycle, whereas continued PD-1 blockade may help sustain newly activated tumor-reactive T-cell responses. To illustrate this concept, we present two cases of recurrent oral cancer treated with the ICI–PIT–ICI sequence. Both patients achieved durable clinical and radiological complete responses following PIT and subsequent nivolumab continuation. Longitudinal analyses of peripheral immune surrogate markers demonstrated a biphasic temporal pattern characterized by transient increases in inflammatory markers, including neutrophil-to-lymphocyte ratio, C-reactive protein, platelet-to-lymphocyte ratio, and systemic immune-inflammation index, followed by recovery trends in absolute lymphocyte count and lymphocyte-to-monocyte ratio during continued PD-1 blockade. These observations support the biological plausibility of PIT as an immune-modulating intervention with potential immune-reprogramming effects. Although hypothesis-generating, the ICI–PIT–ICI sequence may represent a promising strategy integrating locoregional tumor destruction with systemic immune modulation in recurrent oral cancer. Further prospective studies incorporating peripheral and tissue-based immune profiling are warranted.

## 1. Introduction

Immune checkpoint inhibitors (ICIs) targeting the PD-1/PD-L1 axis have significantly changed the treatment landscape for recurrent or metastatic head and neck squamous cell carcinoma (HNSCC), including oral squamous cell carcinoma (OSCC) [1,2,3]. However, durable responses remain limited, and many patients eventually develop resistance or disease progression after systemic therapy. Therefore, novel treatment strategies capable of restoring or sustaining antitumor immunity are needed.

Near-infrared photoimmunotherapy (PIT) has emerged as a tumor-selective locoregional treatment for unresectable or recurrent HNSCC [4,5,6,7,8]. PIT induces rapid and selective tumor cell death through near-infrared light activation of cetuximab sarotalocan sodium bound to epidermal growth factor receptor-expressing tumor cells [4,5,6,7,8,9,10,11]. In addition to direct cytotoxicity, PIT has been shown to induce immunogenic cell death, enhance antigen presentation, and stimulate host antitumor immunity [4,5,10,12,13]. These immunological effects suggest that PIT may function not only as a local ablative therapy but also as an immune-modulating intervention.

The oral cavity is a particularly important setting for PIT because recurrent oral cancers often arise in previously treated fields where salvage surgery may cause severe functional impairment. Recent real-world analyses demonstrated favorable local control of oral-site lesions treated with PIT while emphasizing the need for multidisciplinary supportive care [14]. Furthermore, several preclinical and clinical studies have suggested that PIT may synergize with PD-1 blockade and potentially restore sensitivity to ICIs [10,13,15,16,17,18]. At the same time, the clinical indications for PD-1 blockade have continued to expand, including perioperative treatment for locally advanced HNSCC, highlighting the growing role of immunotherapy across different stages of disease management [19,20,21].

Recent advances in tumor immunology have demonstrated that durable responses to PD-1 blockade depend on precursor exhausted T cells, tumor-draining lymph nodes, and sustained T-cell priming within the cancer-immunity cycle [22,23,24,25,26,27,28,29,30,31,32,33,34,35,36,37,38,39,40,41,42]. In this article, we use the term “immune-reset” as a conceptual framework to describe PIT-induced tumor antigen release and inflammatory reactivation that may reinitiate elements of the cancer-immunity cycle, rather than as direct evidence of complete immunological reprogramming. Within this framework, PIT may exert an immune-reset-like effect, whereas subsequent continuation or re-administration of ICIs may help sustain antitumor immune responses.

In this review, we summarize current evidence regarding PIT for oral-site HNSCC and discuss the immunological significance of sequential ICI–PIT–ICI therapy (ICI–PIT–ICI sequence). We additionally present our institutional experience and longitudinal analyses of peripheral immune surrogate markers, including absolute lymphocyte count (ALC), neutrophil-to-lymphocyte ratio (NLR), lymphocyte-to-monocyte ratio (LMR), platelet-to-lymphocyte ratio (PLR), C-reactive protein (CRP), and systemic immune-inflammation index (SII), as hypothesis-generating indicators of treatment-induced immune dynamics. The novelty of this review does not lie in proposing PIT–ICI combination therapy itself, which has been previously reported, but rather in conceptualizing PIT within a sequential ICI–PIT–ICI framework and integrating illustrative clinical observations with longitudinal peripheral immune surrogate analyses. Based on these observations, we propose a hypothesis-generating biphasic model consisting of transient inflammatory activation after PIT followed by a shift toward a more favorable peripheral immune profile during continued PD-1 blockade. This conceptual framework is intended to generate biological hypotheses and should not be interpreted as evidence of direct immune reprogramming or mechanistic validation.

This article was conducted as a narrative review. Relevant literature was identified through searches of PubMed and Scopus using combinations of keywords including “photoimmunotherapy”, “near-infrared photoimmunotherapy”, “immune checkpoint inhibitor”, “oral squamous cell carcinoma”, “head and neck squamous cell carcinoma”, “immunogenic cell death”, and “cancer immunology”. Publications published between January 2015 and May 2026 were primarily considered, while seminal earlier studies were included when relevant. Additional references were identified through manual review of the bibliographies of selected articles. Because of the narrative nature of this review, no formal systematic review methodology, predefined inclusion/exclusion criteria, or meta-analysis was performed.

## 2. Current Clinical Role of PIT in Oral Cancer

PIT has emerged as a novel locoregional treatment option for recurrent or unresectable HNSCC, particularly in patients with limited salvage options after surgery, radiotherapy, or systemic therapy [4,5,6,7,8]. In Japan, cetuximab sarotalocan sodium combined with near-infrared light irradiation has been clinically introduced for unresectable locally advanced or recurrent disease, and PIT is increasingly incorporated into multidisciplinary management strategies [26,43].

Among HNSCC subsites, the oral cavity represents a unique and clinically important setting for PIT. Recurrent oral cancers frequently arise in previously treated fields, where additional surgery may result in substantial functional morbidity involving speech, mastication, swallowing, and facial appearance. Therefore, minimally invasive local therapies capable of preserving oral function are particularly valuable in this population.

Recent real-world studies have demonstrated encouraging outcomes of PIT for oral-site lesions. Hasegawa et al. analyzed multicenter data focusing on oral-site PIT and reported meaningful local tumor control with acceptable safety profiles [14]. Similarly, Tahara et al. reported favorable clinical outcomes in recurrent or unresectable HNSCC treated with PIT, including patients with oral cavity lesions [44]. These reports support the feasibility of PIT as a local salvage modality in heavily treated patients (Table 1).

However, clinical management of oral-site PIT requires careful multidisciplinary support. Treatment-associated edema, pain, mucositis, bleeding, dysphagia, nutritional deterioration, and airway compromise are particularly important considerations in oral cancer patients [14,44]. Accordingly, PIT for oral cancer should not be regarded as a simple outpatient local procedure, but rather as part of comprehensive supportive and oncologic care.

Importantly, the role of PIT may extend beyond local cytoreduction. Increasing evidence suggests that PIT can induce immunogenic cell death and activate systemic antitumor immunity through tumor antigen release and inflammatory signaling [4,5,12]. Several preclinical and clinical studies have further suggested synergistic interactions between PIT and immune checkpoint blockade [10,13,15,16,17,18]. These findings raise the possibility that PIT may function not only as a local ablative therapy but also as an immunological intervention capable of remodeling the tumor microenvironment and restoring responsiveness to ICIs.

Nevertheless, the optimal integration of PIT into systemic immunotherapy remains unclear. In particular, limited evidence is available regarding sequential treatment strategies in which ICIs are administered before and after PIT. Understanding the immunological significance of this ICI–PIT–ICI sequence may therefore be important for improving durable disease control in recurrent oral cancer.

Despite these encouraging findings, the current clinical evidence supporting PIT in recurrent oral cancer remains limited. Most available studies consist of retrospective analyses, single-center experiences, subgroup analyses, or small observational cohorts, and many include highly selected patients treated at specialized institutions [14,18,44]. Consequently, reported outcomes may be influenced by selection bias, institutional expertise, and heterogeneity in patient characteristics and treatment protocols. Furthermore, comparative data against established treatment strategies remain scarce, and long-term oncologic outcomes have not been fully characterized. Therefore, although existing studies support the feasibility and potential clinical utility of PIT, the overall quality of evidence remains limited, and prospective multicenter studies are required to better define its role in recurrent oral cancer.

## 3. Immunological Basis of PIT

PIT exerts antitumor effects through both direct tumor cell destruction and activation of host immunity [4,5,6,11,12]. Following binding of cetuximab sarotalocan sodium to epidermal growth factor receptor (EGFR)-expressing tumor cells, near-infrared light exposure induces rapid membrane disruption and selective necrotic cell death [6,8]. Unlike conventional thermal ablation, PIT preserves surrounding stromal structures while inducing highly immunogenic tumor destruction [5,12]. Importantly, PIT has been shown to induce immunogenic cell death (ICD), resulting in the release of tumor-associated antigens and danger-associated molecular patterns, including calreticulin, ATP, and HMGB1 [4,5,12]. These signals promote dendritic cell maturation, antigen presentation, and subsequent activation of tumor-specific T cells, thereby contributing to re-initiation of the cancer-immunity cycle [42,45]. Preclinical studies have demonstrated increased CD8-positive T-cell infiltration and enhanced systemic antitumor immunity following PIT [5,10,13].

Recent advances in tumor immunology have highlighted the importance of precursor exhausted T cells (TPEX), tumor-draining lymph nodes (tdLNs), and tertiary lymphoid structures in mediating durable responses to PD-1 blockade [32,33,34,35,36,37,38,39,40,41]. However, these mechanisms were not directly evaluated in the present cases, and their specific roles in recurrent oral cancer treated with PIT remain uncertain. Emerging evidence from experimental and translational studies suggests that durable responses to PD-1 blockade may depend on stem-like tumor-reactive T-cell populations and sustained antigen presentation within tdLNs [29,32,33,34,35,36,37,38]. Therefore, these concepts are discussed here as a biologically plausible framework for interpreting potential interactions between PIT and PD-1 blockade rather than as mechanisms demonstrated in our patients.

Within this conceptual framework, PIT-induced immunogenic cell death may provide a renewed source of tumor antigens and inflammatory signals that could potentially support reactivation of antitumor immunity. Similarly, continued PD-1 blockade after PIT may help sustain newly primed or reactivated tumor-reactive T-cell populations. Experimental studies have demonstrated synergistic antitumor effects of PIT and PD-1 blockade, including enhanced immune activation, increased immune-cell infiltration, and improved systemic tumor control in preclinical models [4,13,17]. Clinically, several case reports and retrospective studies have suggested that continuation or re-administration of ICIs after PIT may be associated with durable tumor control in selected patients with recurrent HNSCC [10,15,16,18]. However, the underlying immunological mechanisms remain incompletely understood, and no direct evidence of TPEX modulation, tdLN activation, immune reprogramming, or tumor microenvironment remodeling was obtained in the present cases. Accordingly, the ICI–PIT–ICI sequence may represent a clinically relevant treatment strategy that is increasingly being adopted in selected patients with recurrent oral cancer, despite the current lack of definitive mechanistic evidence supporting its use. Emerging clinical observations, including the present cases, suggest that this sequential approach may contribute to durable disease control in some patients. However, the proposed immunological mechanisms remain largely conceptual and hypothesis-generating at present. Future translational and prospective studies integrating clinical outcomes with detailed immune profiling will be essential to determine whether the ICI–PIT–ICI sequence can evolve from a biologically plausible concept into a scientifically validated and optimized treatment strategy.

## 4. Synergistic Effects of the ICI–PIT–ICI Sequence for Recurrent Oral Cancer

**Figure 1:**
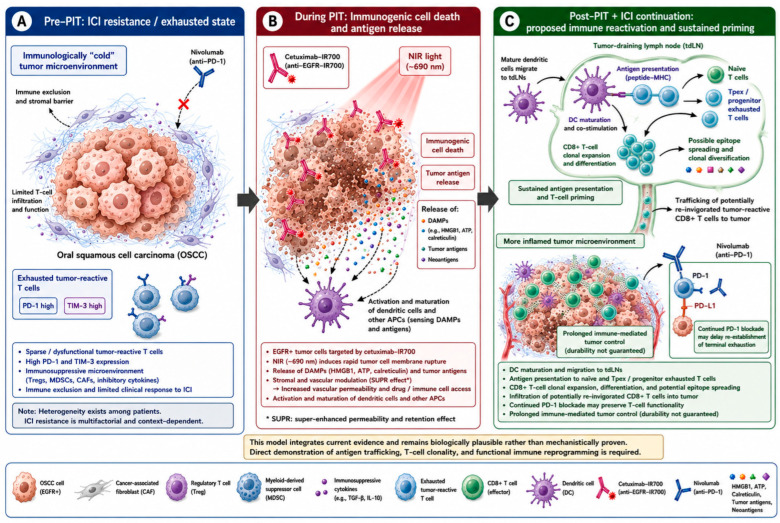
Conceptual framework of the proposed immunological interactions during the ICI–PIT–ICI sequence in recurrent oral cancer. Pre-treatment PD-1 blockade may partially activate tumor-reactive T cells but may eventually become insufficient because of persistent antigen exposure and progressive immune dysfunction (**A**). PIT induces immunogenic cell death and release of tumor-associated antigens and danger-associated molecular patterns (**B**). Continued PD-1 blockade after PIT may potentially support antitumor immunity through mechanisms proposed in the immunology literature, including renewed antigen presentation and sustained T-cell responses (**C**). The characteristic biphasic changes observed in peripheral immune surrogate markers are also illustrated. Importantly, the immunological events depicted in Panel (**C**) were not directly evaluated in the present cases and are presented as a hypothesis-generating conceptual framework rather than a validated biological pathway.

Despite the clinical benefits of ICIs in recurrent or metastatic HNSCC, many patients ultimately develop primary or acquired resistance [1,2,3,24]. Mechanisms of resistance include insufficient tumor antigen presentation, impaired T-cell priming, immunosuppressive tumor microenvironments, and progressive T-cell exhaustion [22,23,24,25,26,27,28,29,30,31,32,33,34,35,36,37]. In recurrent oral cancer, repeated surgery, radiotherapy, and systemic therapy may further contribute to local immune dysfunction and limited responsiveness to subsequent treatments (Figure 1).

In this context, sequential treatment with ICIs before and after PIT may provide synergistic immunological effects rather than simple additive local control. Preceding ICI therapy may partially activate tumor-reactive T cells and establish an initial immune response, even in clinically refractory disease. However, sustained antigen exposure and chronic inflammation can eventually result in terminal T-cell exhaustion and loss of effective antitumor activity [27,32,33,34,35].

PIT may alter this immune state through induction of ICD. Furthermore, PIT-induced tumor destruction may promote epitope spreading, whereby subdominant or previously concealed tumor antigens are released and subsequently recognized by the immune system. This process may broaden the repertoire of tumor-reactive T cells and allow PD-1 blockade to reinvigorate newly primed or newly recruited T-cell clones, including those not effectively engaged during the initial ICI exposure. Experimental studies have demonstrated that PIT promotes release of tumor-associated antigens and danger-associated molecular patterns, enhances dendritic cell activation, increases CD8-positive T-cell infiltration, and stimulates systemic antitumor immunity [4,5,10,12,13]. Unlike conventional cytotoxic therapies, PIT may function as an “immune-reset-like” intervention with the potential to reinitiate components of the cancer-immunity cycle [42,45].

Importantly, continuation or re-administration of ICIs after PIT may help sustain and amplify this newly activated immune response. Recent immunological studies have shown that durable responses to PD-1 blockade depend on TPEX, stem-like CD8-positive T cells, tdLNs, and continuous T-cell priming [29,32,33,34,35,36,37,38]. PIT-induced antigen release and inflammatory signaling may provide renewed immune stimulation, whereas subsequent ICI therapy may help sustain antitumor immune responses within a biologically plausible immune-remodeling framework, although this mechanism was not directly evaluated in the present cases.

Because tumor-draining lymph nodes serve not only as sites of T-cell priming but may also influence metastatic dissemination, preservation and functional reactivation of these immune niches may have therapeutic relevance in sequential ICI–PIT–ICI therapy, although this concept remains speculative [46,47].

This sequential process may contribute to restoration of antitumor immune sur-veillance against residual or dormant tumor cells [48,49,50,51].

Several clinical observations are consistent with this concept, although they do not establish a causal synergistic effect between PIT and ICIs. Case reports and retrospective studies have suggested favorable responses when ICIs were administered after PIT in recurrent HNSCC [10,15,16,18]. Furthermore, combination studies of PIT and pembrolizumab demonstrated the feasibility of integrating PIT with PD-1 blockade in heavily treated patients [17]. Although definitive clinical evidence remains limited, these findings support the biological plausibility of the ICI–PIT–ICI sequence as a strategy for immune remodeling in recurrent oral cancer.

The present cases demonstrated characteristic longitudinal changes in peripheral immune surrogate markers following PIT during ongoing ICI treatment. However, because both patients continued nivolumab before and after PIT, the relative contributions of PIT, ongoing PD-1 blockade, delayed immunotherapeutic effects, and individual patient factors cannot be determined. Importantly, the observations presented in this review do not allow definitive attribution of clinical outcomes to a synergistic interaction between PIT and PD-1 blockade. It remains possible that durable responses were influenced by delayed effects of nivolumab, favorable underlying tumor biology, or other patient-specific factors. Furthermore, because no comparator group receiving continued ICI therapy without PIT was available, the incremental contribution of PIT cannot be quantified. Therefore, the proposed synergistic model should be regarded as a biologically plausible and clinically informed hypothesis rather than direct evidence of treatment synergy. In both cases, transient elevations in inflammatory markers, including NLR, CRP, and SII, were observed shortly after PIT, followed by subsequent decreases accompanied by recovery trends in ALC and LMR. Although these findings may partly reflect tumor burden reduction, the biphasic temporal pattern may be consistent with transient treatment-related inflammatory activation followed by partial recovery of systemic immune status. These observations are consistent with, but do not establish, treatment-related immune modulation during continued ICI therapy.

Taken together, sequential ICI–PIT–ICI therapy may represent a clinically relevant treatment strategy that warrants further investigation. Although emerging clinical observations support its potential utility, the proposed immunological synergy and immune-remodeling effects remain hypothetical and require validation in prospective translational studies.

## 5. Illustrative Cases of the ICI–PIT–ICI Sequence

To illustrate the clinical and immunological relevance of this concept, we present two representative cases of recurrent oral cancer treated with sequential ICI–PIT–ICI therapy. These cases were not intended to establish efficacy or demonstrate treatment synergy. Rather, they are presented as illustrative clinical observations intended to generate hypotheses regarding potential treatment-induced immune dynamics and the feasibility of the ICI–PIT–ICI sequence.

Case 1

**Figure 2:**
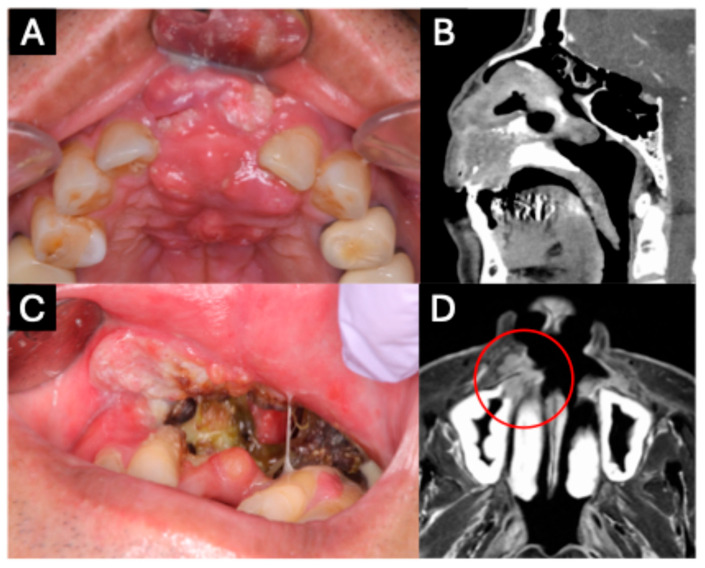
Clinical and radiological findings of the primary and recurrent tumors. (**A**) Clinical photograph of the primary tumor arising from the right maxillary gingiva. (**B**) Contrast-enhanced CT image demonstrating extension of the primary lesion into the nasal cavity with destruction of the anterior nasal spine. (**C**) Clinical photograph of the recurrent lesion extending from the right gingivobuccal sulcus to the inner surface of the upper lip. (**D**) Contrast-enhanced MRI demonstrating recurrent tumor involvement of the nasal cavity and anterior wall of the maxillary sinus (red circle).

**Figure 3:**
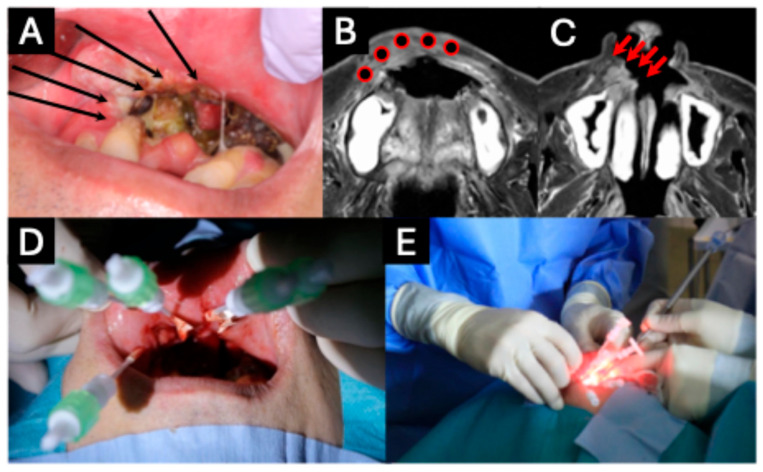
Preoperative planning and intraoperative findings of PIT. (**A**) Preoperative clinical photograph demonstrating the recurrent lesion involving the upper lip and maxillary gingival region. (**B**) Preoperative MRI used for treatment planning, showing the extent of tumor involvement and the intended illumination area. (**C**) Schematic and clinical planning of needle catheter placement. Because PIT carried a potential risk of cutaneous fistula formation in the upper lip, the upper lip was manually everted and the catheters were inserted through the central thickness of the lip to avoid direct penetration of the skin surface. Clinical and MRI findings were used to determine the illumination field and catheter positions (arrows and circles). (**D**) Intraoperative photograph showing placement of multiple needle catheters before light irradiation. (**E**) Intraoperative photograph demonstrating near-infrared light irradiation during PIT using cylindrical diffusers.

A 59-year-old man with recurrent maxillary gingival squamous cell carcinoma developed platinum-resistant local recurrence after induction chemotherapy and definitive chemoradiotherapy (Figure 2). Nivolumab was initiated; however, persistent locally recurrent disease involving the upper gingiva, upper lip mucosa, nasal cavity, and maxillary sinus remained after 10 courses. he patient experienced worsening local pain and persistent symptomatic disease burden. Therefore, despite evidence of partial local response, nivolumab alone was considered insufficient for local disease control, and PIT was selected to achieve more effective locoregional management while continuing PD-1 blockade. PIT was therefore selected as an additional locoregional intervention during continued PD-1 blockade and performed under general anesthesia using cylindrical and side-fire diffusers (Figure 3).

**Figure 4:**
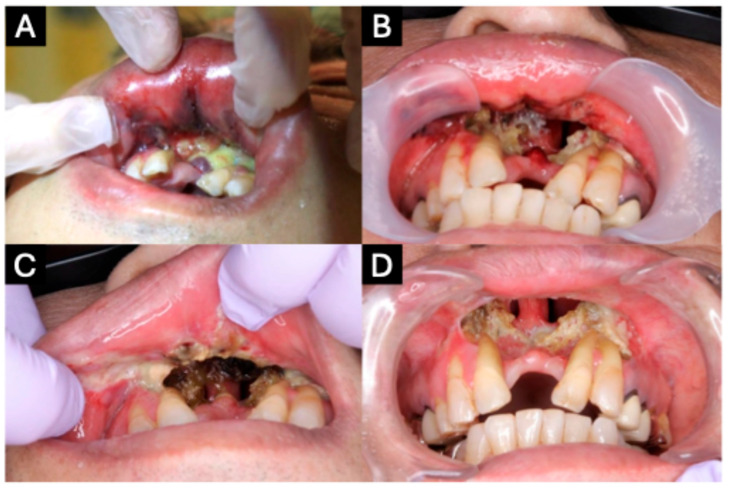
Postoperative clinical course after PIT. (**A**): Marked upper lip edema and dark-purple discoloration of the oral mucosa were observed on the day after PIT. (**B**): Progressive necrosis developed within 1 week after PIT, and necrotic tissue was subsequently debrided. (**C**): Two weeks after PIT, the necrotic tumor had sloughed off, with surrounding exposed sequestrum observed. (**D**): At 4 weeks after PIT, the wound surface had become flattened. The patient has maintained complete response for more than 6 months after PIT.

Marked post-treatment edema and tumor necrosis developed, followed by spontaneous tumor sloughing and repeated debridement (Figure 4). ICI was resumed 4 weeks after PIT and has been continued thereafter. The patient has maintained clinical and radiological complete response, with no detectable recurrent lesions on follow-up examination and imaging for 10 months after PIT. Longitudinal analyses of peripheral immune surrogate markers demonstrated characteristic temporal changes during the ICI–PIT–ICI sequence. During the acute post-PIT phase, transient increases in inflammatory markers, including NLR, CRP, and SII, were observed, likely reflecting treatment-related inflammatory activation associated with tumor necrosis and tissue injury. Subsequently, these inflammatory indices decreased during continued nivolumab therapy, accompanied by recovery trends in ALC and LMR (Table 2). Rather than indicating a unidirectional improvement, these biomarker dynamics were interpreted as a biphasic response, consisting of an early inflammatory phase followed by a later shift toward a more favorable peripheral immune profile during continued PD-1 blockade. Given the single-patient, repeated-measure nature of these data, these observations were interpreted descriptively rather than as confirmatory statistical evidence.

Peripheral immune surrogate markers were evaluated across three predefined treatment phases: (1) the pre-PIT ICI treatment period after recurrence diagnosis, (2) the first 4 weeks after PIT, corresponding to the anticipated acute inflammatory phase, and (3) the period beyond 4 weeks post-PIT during resumed ICI therapy. Mean values for each phase are presented descriptively to illustrate temporal biomarker dynamics. Because these analyses were based on repeated longitudinal measurements in two illustrative cases, no formal inferential statistical testing was performed.

Case 2

A 47-year-old man with recurrent tongue squamous cell carcinoma experienced multiple local recurrences despite multimodal treatment, including surgery, postoperative chemoradiotherapy, salvage surgery, and cetuximab plus paclitaxel chemotherapy. After development of extensive recurrent lesions involving the gingiva, palate, and buccal mucosa, nivolumab therapy was initiated. After two courses of nivolumab, progressive enlargement of the local lesions was observed. Although delayed responses can occur during PD-1 blockade, further observation without local intervention was considered clinically undesirable because of ongoing tumor progression and the risk of further local deterioration. Therefore, PIT was performed as an additional locoregional treatment while nivolumab therapy was subsequently continued.

**Figure 5:**
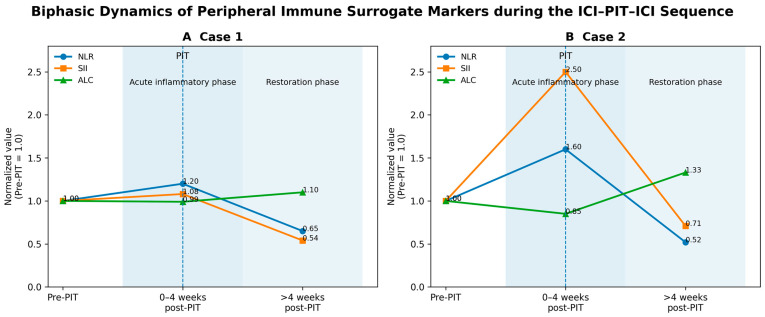
Biphasic dynamics of peripheral immune surrogate markers during the ICI–PIT–ICI sequence. (**A**) **Case 1.** Normalized longitudinal changes in peripheral immune surrogate markers during sequential ICI–PIT–ICI therapy. NLR and SII showed transient increases during the acute post-PIT phase, followed by decreases during continued nivolumab therapy, whereas ALC remained stable initially and subsequently increased during the restoration phase. (**B**) **Case 2.** Normalized longitudinal changes in peripheral immune surrogate markers during sequential ICI–PIT–ICI therapy. NLR and SII demonstrated more pronounced transient elevations during the acute post-PIT phase, followed by marked decreases during continued nivolumab therapy. In contrast, ALC decreased transiently after PIT and subsequently recovered during the restoration phase. **Values were normalized to the pre-PIT baseline (set as 1.0) to illustrate temporal biomarker trajectories across treatment phases. These observations are descriptive and hypothesis-generating and should not be interpreted as evidence of treatment-induced immune reprogramming. Abbreviations:** ALC, absolute lymphocyte count; ICI, immune checkpoint inhibitor; NLR, neutrophil-to-lymphocyte ratio; PIT, photoimmunotherapy; SII, systemic immune-inflammation index.

Progressive tumor necrosis and mucosal healing were observed after PIT. Nivolumab therapy was resumed 4 weeks after PIT and has been continued thereafter. The patient has maintained clinical and radiological complete response, with no detectable recurrent lesions on follow-up examination and imaging for 7 months after PIT. Similar to Case 1, longitudinal peripheral immune surrogate analyses demonstrated a biphasic temporal pattern during the ICI–PIT–ICI sequence. Inflammatory markers transiently increased during the acute post-PIT period, followed by subsequent decreases during continued nivolumab treatment, accompanied by recovery trends in ALC and LMR (Figure 5). Although interpretation is limited by the small number of repeated measurements in a single case, the overall trajectory was consistent with transient treatment-related inflammatory activation followed by a later shift toward a more favorable peripheral immune profile (Figure 5).

Table 1 (A) and Case 2 (B). Values were normalized to pre-PIT levels (set as 1.0) to facilitate comparison across treatment phases. NLR and SII demonstrated transient increases during the acute post-PIT phase, consistent with treatment-related inflammatory activation, followed by decreases during continued nivolumab therapy. In contrast, ALC showed recovery trends during the restoration phase. These temporal patterns are consistent with a biphasic response characterized by early inflammatory activation followed by a later shift toward a more favorable peripheral immune profile during continued PD-1 blockade. These findings should be interpreted descriptively because they are derived from two illustrative cases and do not establish causal immunological mechanisms. Raw biomarker values corresponding to the normalized trajectories are provided in Table 2.

## 6. Peripheral Immune Surrogates

Identification of clinically applicable biomarkers remains an important challenge in the development of the ICI–PIT–ICI sequence for recurrent oral cancer. Although comprehensive immunological analyses using tumor tissue, spatial profiling, and single-cell technologies are increasingly feasible, these approaches are not always practical in routine clinical settings. Therefore, peripheral immune surrogate markers obtained from routine blood examinations may represent useful and minimally invasive tools for longitudinal immune monitoring.

However, these biomarkers should be interpreted with caution. Parameters such as NLR, PLR, LMR, SII, CRP, and ALC are nonspecific and may be influenced by multiple biological and clinical factors, including treatment-related inflammation, tissue injury, tumor necrosis, infection, wound healing, systemic stress responses, and changes in tumor burden. Consequently, temporal changes in these markers cannot be regarded as direct evidence of immune reprogramming or specific immunological mechanisms. Rather, they should be considered descriptive surrogate indicators that may provide indirect insights into systemic immune and inflammatory dynamics during treatment.

Among these markers, NLR has been widely associated with systemic inflammation, tumor progression, and poor prognosis across multiple malignancies, including HNSCC [1,2,3,52,53,54,55]. Elevated NLR is generally considered to reflect neutrophil-mediated immunosuppression and impaired lymphocyte-dependent antitumor immunity. In patients with recurrent or metastatic HNSCC treated with ICIs, post-treatment NLR dynamics have also been associated with therapeutic response and survival outcomes [52,53]. Similarly, SII calculated using platelet, neutrophil, and lymphocyte counts, has emerged as a composite biomarker reflecting systemic inflammatory and immune status. High SII values have been associated with inferior oncologic outcomes and reduced responsiveness to immunotherapy [52,53,54,55].

ALC and LMR may also provide clinically relevant information regarding host immune competence. Lymphocytes play central roles in tumor-specific immune responses, whereas monocytes may contribute to immunosuppressive tumor-associated macrophage populations. Accordingly, low ALC and low LMR have been associated with impaired antitumor immunity and unfavorable prognosis in HNSCC and other solid tumors [52,53,54,55]. PLR is another inflammatory biomarker linked to tumor progression, angiogenesis, and immune evasion [52,53,54,55]. In addition, CRP is a well-established marker of systemic inflammation and may reflect treatment-related inflammatory responses following PIT.

In these illustrative cases, characteristic temporal changes in these peripheral immune surrogate markers were observed during the ICI–PIT–ICI sequence. Rather than representing a unidirectional improvement, these biomarker dynamics may be more appropriately interpreted as a biphasic immune response. During the acute post-PIT phase, transient increases in NLR, CRP, PLR, and SII were observed, likely reflecting an early innate inflammatory phase driven by local tumor necrosis, tissue injury, neutrophil recruitment, and macrophage-mediated clearance after PIT. Subsequently, these inflammatory indices decreased during continued PD-1 blockade, accompanied by recovery trends in ALC and LMR. This later phase may reflect a relative shift toward a more favorable systemic immune profile characterized by partial recovery of lymphocyte-mediated adaptive immunity. Although these findings may partly reflect reduction in tumor burden, the biphasic temporal pattern may help reconcile the apparently paradoxical early inflammatory spike with subsequent clinical tumor control.

Importantly, peripheral immune surrogate markers may provide indirect insights into treatment-related modulation of the cancer-immunity cycle [42,45]. PIT-induced immunogenic cell death may transiently amplify inflammatory signaling through tumor antigen release, whereas subsequent continuation of ICIs may contribute to maintenance of antitumor immune responses during the proposed immune-reset-like process. Therefore, longitudinal monitoring using routine blood biomarkers may help characterize systemic immune dynamics during the ICI–PIT–ICI sequence, although causal immunological mechanisms cannot be established from peripheral markers alone.

However, peripheral blood biomarkers provide only indirect information regarding the tumor immune microenvironment. Therefore, histopathological and immunological analyses using biopsy or resected specimens obtained before and after PIT may further improve understanding of treatment-induced immune modulation. In particular, evaluation of CD8-positive T-cell infiltration, FOXP3-positive regulatory T cells, CD8/FOXP3 ratio, PD-L1 expression, and Granzyme B expression may help characterize the transition from an immunologically “cold” to “hot” tumor microenvironment [31,32,33,34,35,36,37,38,39,40,41,42,45,55]. In addition, assessment of HLA class I and β2-microglobulin expression may provide further insights into antigen presentation capacity and mechanisms of immune responsiveness or resistance during the ICI–PIT–ICI sequence. Future translational studies integrating peripheral immune surrogate markers with tissue-based immunological analyses, spatial immune profiling, and T-cell receptor repertoire analyses are warranted.

## 7. Clinical Implications and Future Perspectives

The present review highlights the potential clinical significance of the ICI–PIT–ICI sequence as an immune-oriented treatment strategy for recurrent oral cancer. Traditionally, PIT has primarily been regarded as a locoregional salvage modality for unresectable or recurrent HNSCC [4,5,6,7,8]. However, accumulating experimental and clinical evidence suggests that PIT may exert broader immunological effects through induction of immunogenic cell death, activation of antigen presentation, and enhancement of tumor-specific T-cell responses [5,10,12,13,16,17]. Accordingly, PIT should not be considered solely as a cytoreductive local therapy, but also as a possible trigger for immune remodeling.

In this context, continuation or re-administration of ICIs after PIT may be particularly important. PIT-induced tumor antigen release and inflammatory activation may transiently enhance antitumor immune responses, whereas subsequent PD-1 blockade may help sustain these responses. The characteristic biphasic temporal patterns observed in peripheral immune surrogate markers are consistent with this hypothesis, although definitive mechanistic conclusions cannot be drawn from two illustrative cases alone. Importantly, the ICI–PIT–ICI sequence may have unique relevance in recurrent oral cancer because oral lesions are often accessible for direct visualization, repeated biopsy, and local intervention, allowing longitudinal clinical and translational evaluation. Furthermore, preservation of oral function and quality of life remains a major therapeutic goal in heavily treated patients who are poor candidates for extensive salvage surgery.

Nevertheless, several important questions remain unresolved. The optimal timing and duration of ICI administration before and after PIT are currently unknown. Similarly, patient selection criteria, ideal treatment sequencing, and predictors of durable response remain unclear. Because treatment-related inflammation itself may influence peripheral immune biomarkers, interpretation of longitudinal immune surrogate changes should be performed cautiously. Safety considerations should also be acknowledged when applying the proposed ICI–PIT–ICI sequence. Although immune checkpoint inhibitors are generally well tolerated, immune-related adverse events, including cardiovascular toxicities such as myocarditis, pericarditis, and arrhythmias, may occur and require appropriate monitoring during continued or repeated ICI administration. Prospective studies with larger cohorts are therefore necessary to validate the clinical significance of these findings. Future translational investigations integrating peripheral biomarkers with tissue-based immune profiling may help clarify the biological mechanisms associated with the ICI–PIT–ICI sequence and identify biomarkers predictive of durable clinical benefit.

Ongoing prospective trials may further clarify the role of PIT within multimodal immunotherapy strategies. Although the design differs from the proposed ICI–PIT–ICI sequence described in the present review, the ongoing phase III ECLIPSE study is evaluating ASP-1929 photoimmunotherapy in combination with pembrolizumab as first-line treatment compared with standard-of-care therapy in patients with locoregionally recurrent HNSCC without distant metastases [55]. The results of this trial may provide important evidence regarding whether PIT can be integrated with PD-1 blockade as part of a broader immune-modulating treatment strategy and may help inform future optimization of PIT-based immunotherapy, including sequential approaches.

## 8. Conclusions

PIT represents a promising treatment modality for recurrent oral cancer not only through local tumor destruction but also through potential activation of host antitumor immunity. Emerging evidence suggests that ICI–PIT–ICI sequence may provide immunological synergy by combining pre-existing immune priming, PIT-induced immunogenic cell death, and sustained immune activation through continued PD-1 blockade. In the illustrative cases presented in this review, longitudinal peripheral immune surrogate analyses demonstrated characteristic temporal changes consisting of transient inflammatory activation after PIT followed by a shift toward a more favorable peripheral immune profile during continued ICI therapy. Although these observations remain hypothesis-generating, they are consistent with the biological plausibility of immune modulation during the ICI–PIT–ICI sequence.

Further prospective and translational studies integrating peripheral biomarkers with tissue-based immunological analyses are warranted to clarify the optimal role of PIT within multimodal immunotherapy strategies for recurrent oral cancer. These findings should be considered hypothesis-generating and warrant validation in prospective translational studies integrating peripheral and tissue-based immune analyses.

## Figures and Tables

**Table 1:** Published reports of PIT for oral cancer with peri-PIT immune checkpoint inhibitor administration.

Study	Design	Number of Cases	Tumor Site	ICI Timing Relative to PIT	Main Outcome	ORR (%)	Survival Outcome
Tsukahara_2024 [16]	Case report	1	HNSCC	ICI after PIT	Durable CR achieved after PIT	NA	CR maintained
Hirakawa_2024 [15]	Retrospective feasibility study	5	Unresectable HNSCC	Concurrent/peri-PIT ICI	Feasible and manageable combination therapy	NA	Not reported
Hasegawa_2026 [14]	Multicenter retrospective subgroup analysis	12	Oral-site HNSCC	Some patients received ICI before and/or after PIT	Favorable local control	83.3	Median OS 22.0 months
Tahara_2026 [44]	Single-center retrospective study	5	Recurrent HNSCC	Some patients received peri-PIT ICI	Favorable local control and survival	NA	Prolonged survival reported
Suzuki_2026	Illustrative cases	2	Recurrent oral SCC	ICI → PIT → ICI	Durable CR with longitudinal immune monitoring	100 (2/2)	CR maintained

PIT, photoimmunotherapy; ICI, immune checkpoint inhibitor; HNSCC, head and neck squamous cell carcinoma; SCC, squamous cell carcinoma; CR, complete response; ORR, objective response rate; OS, overall survival; NA, not available.

**Table 2:** Peripheral immune surrogate dynamics during the ICI–PIT–ICI sequence.

	Pre-PIT	First 4 Weeks Post-PIT	>4 Weeks Post-PIT
Case.1			
ALC	1460	1444	1605
NLR	4.15	4.98	2.69
LMR	2.70	2.37	3.37
PLR	191.2	179.6	148.6
CRP	1.44	1.80	0.18
SII	1,175,168	1,272,695	637,068
Case.2			
ALC	1240	1051	1647
NLR	4.92	7.88	2.56
LMR	4.93	3.13	4.62
PLR	200.3	320.0	159.6
CRP	0.97	0.52	0.18
SII	934,890	2,339,823	667,559

## Data Availability

The data presented in this study are available from the corresponding author upon reasonable request. The data are not publicly available due to privacy and ethical restrictions.

## Abbreviation

PIT	Photoimmunotherapy
ICI	Immune checkpoint inhibitor
HNSCC	Head and neck squamous cell carcinoma
OSCC	Oral squamous cell carcinoma
ICD	Immunogenic cell death
ALC	Absolute lymphocyte count
NLR	Neutrophil-to-lymphocyte ratio
LMR	Lymphocyte-to-monocyte ratio
PLR	Platelet-to-lymphocyte ratio
SII	Systemic immune-inflammation index
CRP	C-reactive protein
tdLN	Tumor-draining lymph node
TPEX	Precursor exhausted T cell
